# Metal-Free Electrochemical Dopamine Sensing Using a g-C_3_N_4_/Polymethyl Thymol Blue Nanohybrid

**DOI:** 10.3390/bios16020124

**Published:** 2026-02-17

**Authors:** Sankar Sekar, Sejoon Lee, Sutha Sadhasivam, Kumar Sangeetha Selvan, Saravanan Sekar, Youngmin Lee, Pugazhendi Ilanchezhiyan, Seung-Cheol Chang, Ramalingam Manikandan

**Affiliations:** 1 Division of System Semiconductor, Dongguk University-Seoul, Seoul 04620, Republic of Korea; sanssekar@dongguk.edu (S.S.); ymlee@dongguk.edu (Y.L.); 2 Quantum-Functional Semiconductor Research Center, Dongguk University-Seoul, Seoul 04620, Republic of Korea; 3 Department of Chemistry, CMS College of Engineering, Namakkal 637003, Tamil Nadu, India; suthaasridhar@gmail.com; 4 Department of Chemistry, VelTech Rangarajan Dr. Sagunthala R&D Institute of Science and Technology, Avadi, Chennai 600062, Tamil Nadu, India; msc.sangi@gmail.com; 5 Department of Mechanical Engineering, K. Ramakrishnan College of Technology, Trichy 621112, Tamil Nadu, India; nanosaran007@gmail.com; 6 Centre for Research and Development, Sri Manakula Vinayagar Engineering College, Madagadipet, Puducherry 605107, India; ilanchezhiyan@gmail.com; 7 Department of Physics, Sri Manakula Vinayagar Engineering College, Madagadipet, Puducherry 605107, India; 8 Department of Cogno-Mechatronics Engineering, College of Nanoscience and Nanotechnology, Pusan National University, Busan 46241, Republic of Korea; 9 Engineering Research Center for Color-Modulated Extra-Sensory Perception Technology, Pusan National University, Busan 46241, Republic of Korea

**Keywords:** graphitic carbon nitride, methyl thymol blue, screen printed carbon electrode, dopamine, biofluids samples

## Abstract

We report a highly sensitive and interference-free electrochemical sensor for dopamine (DA) detection in the presence of uric acid (UA) and ascorbic acid (AA), based on an in situ deposited graphitic carbon nitride (g-C_3_N_4_) and polymethyl thymol blue (PMTB) nanohybrid modified screen-printed carbon electrode (SPCE). The as-fabricated g-C_3_N_4_/PMTB/SPCE was thoroughly characterized using various physicochemical techniques. The electrochemical behavior of the modified electrode was systematically investigated by cyclic voltammetry (CV) and differential pulse voltammetry (DPV). The g-C_3_N_4_/PMTB/SPCE exhibited excellent electrocatalytic activity toward the selective oxidation of DA under optimized experimental conditions, including pH and scan rate. Interference-free detection of DA in the presence of AA and UA was achieved using DPV and chronoamperometric methods, revealing a wide linear concentration range, an ultralow limit of detection, and high sensitivity. Furthermore, the practical applicability of the proposed sensor was validated by determining DA in artificial biofluid samples, including blood serum, and urine. The recovery results obtained good agreement with those obtained using high-performance liquid chromatography (HPLC), confirming the reliability and accuracy of the developed sensing platform.

## 1. Introduction

Nowadays, the electrochemical detection of catecholamines has gained significant attention due to its importance in biomedical analysis [1,2]. Dopamine (DA) plays a crucial role in various biological processes and functions as a key neurotransmitter in the peripheral and central nervous systems, where it is involved in preventing drug abuse and is closely associated with Parkinson’s disease [3,4]. In addition, DA plays an essential role in regulating cognitive functions such as stress response, behavior, and attention. Dopamine exists as a positively charged organic molecule in biological fluids and brain cells, and abnormal DA concentrations can lead to several neurological and metabolic disorders. Under normal physiological conditions, DA is present in human biofluids at concentrations ranging from 10^−7^ to 10^−3^ mol L^−1^ and coexists with relatively high concentrations of ascorbic acid (AA) and uric acid (UA) [5,6]. The determination of DA has traditionally been carried out using conventional analytical techniques such as spectrophotometry and high-performance liquid chromatography coupled with mass spectrometry (HPLC-MS) [7,8,9]. However, these methods require expensive and sophisticated instrumentation, complex sample preparation and pretreatment steps, and are often time-consuming. In contrast, electrochemical techniques offer several advantages, including high sensitivity, low cost, operational simplicity, and compatibility with automation, miniaturization, and in situ detection. Consequently, electrochemical methods have emerged as reliable and efficient alternatives for accurate DA determination [10,11].

A large proportion of previously reported studies rely on expensive materials and complex electrode modification procedures. In recent years, carbon-based nanomaterials, particularly graphitic carbon nitride (g-C_3_N_4_), have demonstrated outstanding performance in electrochemical applications [12,13,14]. g-C_3_N_4_ consists of covalently bonded carbon and nitrogen atoms arranged in a π-conjugated framework, which imparts excellent thermal and chemical stability, as well as remarkable catalytic activity and unique optical and electronic properties. However, its intrinsic electrical conductivity can be significantly enhanced through strategies such as doping with metal nanoparticles, forming composites with conductive carbon materials, or copolymerization with organic molecules [15,16]. One of the key advantages of g-C_3_N_4_ lies in its sp^2^-hybridized carbon and nitrogen atoms, which provide a high density of active sites, a large effective surface area, and enhanced adsorption of target analytes. In parallel, polymer-based electrodes have been widely employed in electrochemical sensing due to their versatility and functional tenability [17,18]. A major advantage of polymer-modified electrodes is their ability to incorporate auxiliary ions or functional groups that interact selectively with target biomolecules, while simultaneously suppressing electrode surface fouling. This protective behavior, often described as a shielding or barrier effect, improves the stability and selectivity of electrochemical sensors compared to unmodified electrodes.

Methyl thymol blue (MTB) is a well-known complexometric indicator containing carboxylic acid and hydroxyl functional groups, and in this work, it was employed in its polymerized form for DA detection. Although these g-C_3_N_4-_based nanocomposite electrodes exhibit promising sensing performance, their fabrication typically involves multistep material synthesis, composite preparation, and complex electrode modification procedures, including the use of various solvents, prolonged drying times, and challenges related to electrode stability [19,20]. To address these limitations, the present study investigates a facile, in situ fabrication strategy for a highly stable, sensitive, and selective DA sensor based on a g-C_3_N_4_/PMTB nanohybrid coated directly onto a screen-printed carbon electrode (SPCE).

Based on a comprehensive review of both earlier studies and recent advances in neurotransmitter detection, this work proposes a novel nanohybrid material with a network-like morphology composed of g-C_3_N_4_ and polymethyl thymol blue (PMTB). The nanohybrid was fabricated by coating it onto a SPCE through a facile one-step in situ electrodeposition or electropolymerization process, using methyl thymol blue as the monomer. The fabricated nanohybrid sensor was systematically evaluated for the highly selective electrochemical detection of DA in the presence of AA and UA. The sensing performance was thoroughly investigated using differential pulse voltammetry (DPV) and amperometric techniques in various human biofluids samples.

## 2. Result and Discussion

### 2.1. Methyl Thymol Blue Polymerization

The experimental procedure, material characterization and the electrochemical bio sensing electrode preparation and the testing parameters are discussed in the Supporting Information. The electrochemical deposition of g-C_3_N_4_ and the electropolymerization of MTB were carried out following our previously reported procedure, with minor modifications. The corresponding CV responses are presented in Figure S1. The CV profiles exhibited two distinct pairs of redox peaks. The first redox pair appeared at −0.06 V/−0.08 V and 0.08 V/0.16 V, with corresponding peak-to-peak separations (ΔE_p_) of −0.02 V and 0.08 V for the initial and subsequent redox couples, respectively. Upon continued potential cycling, a gradual decrease in the cathodic peak current was observed, indicating progressive polymer growth on the electrode surface [21,22].

The appearance of two redox couples suggests that different potential regions are involved in the electrochemical processes. Redox peaks in the negative potential region are attributed to the anodic oxidation of MTB monomers, whereas the redox peaks observed in the positive potential region correspond to the uniform growth of the polymeric film on the electrode surface. In addition, the voltammetric signal observed at approximately 0.5 V is associated with the formation of anionic radical species. During potential scanning, the hydroxyl (-OH) groups linked to the aromatic naphthalene moieties undergo oxidation, leading to the generation of phenoxy radicals. Subsequently, the polymerized methyl thymol blue is proposed to undergo head-to-tail coupling through hydroxyl groups. This redox behavior is consistent with a reversible transformation between naphthol and naphthoquinone forms, as reported in earlier studies [23,24,25]. A schematic representation of the proposed electropolymerization mechanism of methyl thymol blue is illustrated in Scheme S1.

### 2.2. Analysis of Crystalline Structure and Surface Morphology

**Figure 1:**
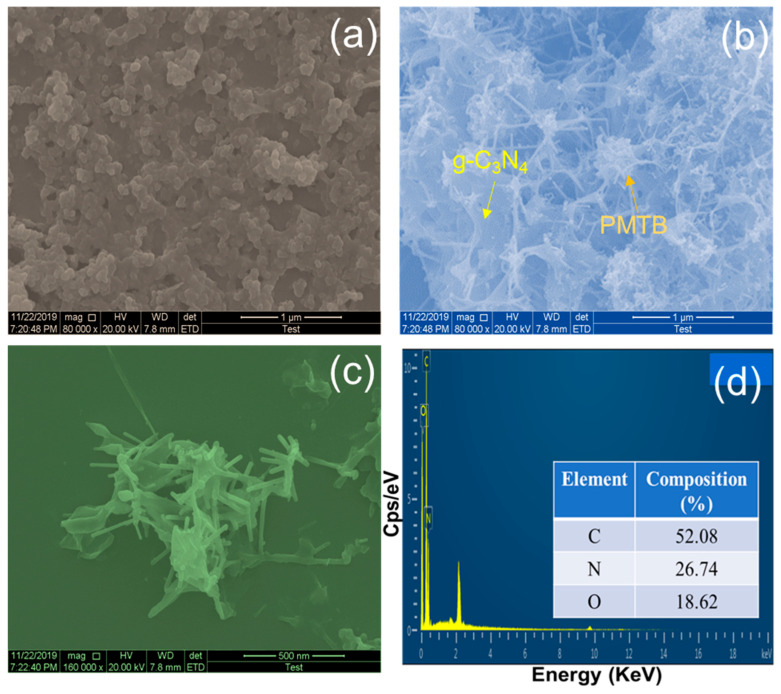
FE-SEM images of (**a**) g-C_3_N_4_, (**b**,**c**) in situ coated nanohybrid of g-C_3_N_4_/PMTB with different magnifications and (**d**) corresponding EDX spectrum.

Figure 1 presents the FE-SEM images of the fabricated g-C_3_N_4_/PMTB/SPCE. As shown in Figure 1a, pristine g-C_3_N_4_ exhibits a thin, flake-like nanosheets morphology [12]. After in situ deposition and electropolymerization, the g-C_3_N_4_/PMTB-modified electrode (Figure 1b) displays a spider-web-like interconnected network structure. The magnified FE-SEM image (Figure 1c) further reveals that the g-C_3_N_4_ nanosheets are uniformly integrated with a stick-like polymer morphology, indicating effective hybridization between g-C_3_N_4_ and PMTB. The corresponding energy-dispersive X-ray (EDX) spectrum (Figure 1d) confirms the elemental composition of the nanohybrid. These FE-SEM observations demonstrate that g-C_3_N_4_ nanosheets are successfully adsorbed and anchored onto the polymerized methyl thymol blue matrix. Consequently, the observed morphological changes clearly confirm the successful in situ deposition of g-C_3_N_4_ and the electropolymerization of methyl thymol blue on the SPCE surface.

**Figure 2:**
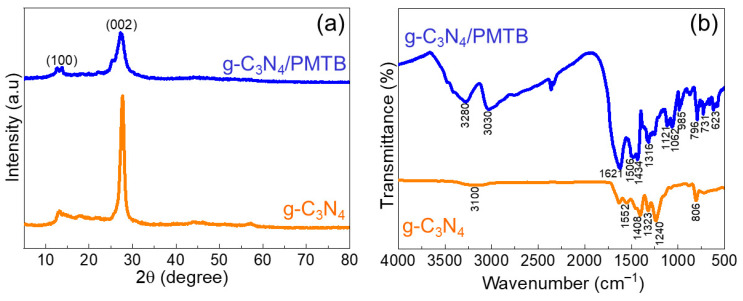
(**a**) XRD pattern and (**b**) FT-IR spectra g-C_3_N_4_ and in situ coated g-C_3_N_4_/PMTB.

The crystalline structure of the prepared g-C_3_N_4_/PMTB nanohybrid was further investigated using X-ray diffraction (XRD) analysis. Figure 2a shows the diffraction patterns of pristine g-C_3_N_4_ and the g-C_3_N_4_/PMTB nanohybrids. The characteristic diffraction peaks observed at 13.22° and 27.42°, corresponding to the (100) and (002) planes, respectively, are attributed to graphitic carbon nitride with a well-ordered layered structure, consistent with the JCPDS card No. 87-1526 [12,17,26]. The prominent peak at 13.22° is associated with the in-plane structural packing of tri-*s*-triazine units within the g-C_3_N_4_ framework [23], as observed in both Figure 2a,b. No additional diffraction peaks are detected after the formation of polymethyl thymol blue, indicating that the polymer layer is largely amorphous and does not disturb the crystalline structure of g-C_3_N_4_. These XRD results confirm the successful formation of the g-C_3_N_4_/PMTB nanohybrid on the electrode surface.

### 2.3. FT-IR Analysis

The functional group vibrations of the as-fabricated g-C_3_N_4_/PMTB/SPCE were investigated using Fourier-transform infrared (FT-IR) spectroscopy. Figure 2b presents the FT-IR spectra of (a) pristine g-C_3_N_4_ and (b) the g-C_3_N_4_/PMTB nanohybrid. For g-C_3_N_4_, the characteristic absorption band observed at 806 cm^−1^ corresponds to the triazine ring breathing mode. The bands appearing at 1240, 1323, 1408, 1552, and 1663 cm^−1^ are attributed to C-N stretching vibrations within the heterocyclic framework [12,17]. In addition, the broad absorption band around 3100 cm^−1^ is assigned to the stretching vibrations of primary and secondary amine groups. After electropolymerization, the g-C_3_N_4_/PMTB nanohybrid exhibits additional characteristic peaks. The absorption bands at 623 and 731 cm^−1^ correspond to C-H stretching vibrations, while the in-plane deformation of C-H stretching is observed at 985 cm^−1^. The triazine breathing mode of g-C_3_N_4_ is retained at 796 cm^−1^, indicating structural integrity after hybrid formation. The peaks at 1121 and 1316 cm^−1^ are attributed to aromatic ring breathing vibrations associated with PMTB. The formation of C-O-C linkages in PMTB is confirmed by the stretching vibration at 1062 cm^−1^, while the bands at 1434 and 1621 cm^−1^ correspond to carboxylic acid stretching vibrations. The C-N stretching vibration of the nanohybrid is observed at 1506 cm^−1^. Furthermore, absorption bands at 3030 and 3280 cm^−1^ are assigned to primary and secondary amine stretching and O-H stretching vibrations, respectively [17,21,22]. These FT-IR results confirm the successful formation of the g-C_3_N_4_/PMTB nanohybrid on the electrode surface.

### 2.4. Electroanalytical Studies

#### 2.4.1. Cyclic Voltammetric Behavior of the g-C_3_N_4_/PMTB/SPCE

**Figure 3:**
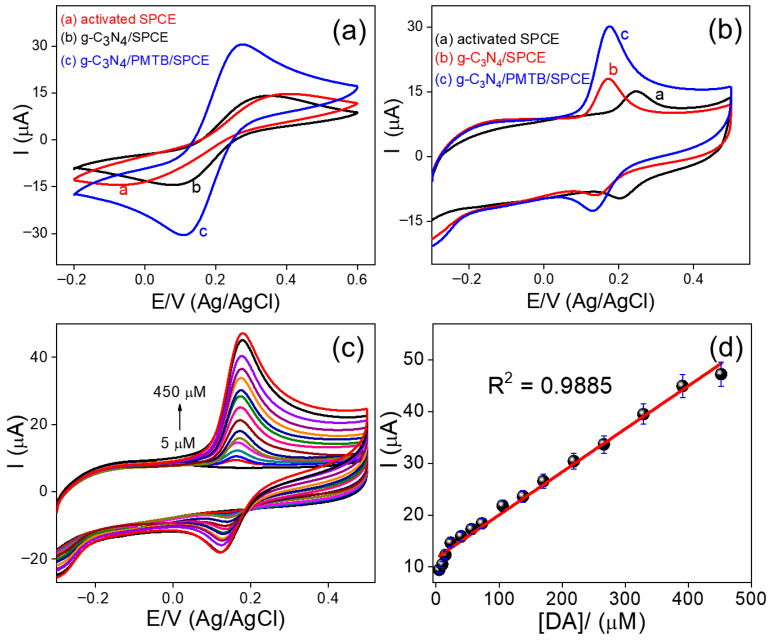
Cyclic voltametric response of (**a**) different kinds of electrodes in 0.05 mM Fe(CN)_6_ in 0.1 M KCl, (**b**) different electrodes in 0.1 M PB solution containing 220 µM of DA, (**c**) g-C_3_N_4_/PMTB nanohybrid different concentration of DA, and (**d**) concentration vs. current plot (concentration range from 5 to 450 µM).

The electrocatalytic activity of the electrochemically prepared g-C_3_N_4_/PMTB nanohybrid modified SPCE was first evaluated using CV. Figure 3a shows the cyclic voltammograms recorded for different electrodes in 0.1 M KCl containing 5 mM [Fe(CN)_6_]^3−/4−^ as a redox probe, within a potential window of −0.2 to 0.6 V at a scan rate of 50 mV/s. The activated SPCE (curve a) exhibits a quasi-reversible redox couple with an anodic peak potential (*E*_pa_) at 0.40 V and a cathodic peak potential (*E*_pc_) at −0.06 V, resulting in a peak-to-peak separation (Δ*E*_p_) of 0.46 V. The g-C_3_N_4_/SPCE (curve b) shows improved electrochemical behavior, with *E*_pa_ at 0.33 V and *E*_pc_ at −0.08 V, yielding Δ*E*_p_ of 0.41 V. In contrast, the g-C_3_N_4_/PMTB/SPCE (curve c) displays a well-defined and nearly reversible redox couple with *E*_pa_ at 0.27 V and *E*_pc_ at 0.10 V, corresponding to a significantly reduced Δ*E*_p_ of 0.17 V. The substantially lower Δ*E*_p_ observed for the g-C_3_N_4_/PMTB/SPCE indicates enhanced electron transfer kinetics compared to the other electrodes. Furthermore, the electrochemically active surface coverage (τ) of the prepared electrodes was estimated using the equation τ = Q/nFA, where τ is the surface coverage (mol cm^−2^), Q is the charge (C), n is the number of electrons transferred, A is the electrode surface area (cm^2^), and F is the Faraday constant (96,485 C mol^−1^). The calculated τ values were 0.23 × 10^−10^, 0.53 × 10^−10^, and 0.87 × 10^−10^ mol cm^−2^ for the activated SPCE, g-C_3_N_4_/SPCE, and g-C_3_N_4_/PMTB/SPCE, respectively. Based on the reduced Δ*E*_p_ values, enhanced redox peak currents, and the significantly increased active surface coverage, the g-C_3_N_4_/PMTB/SPCE demonstrates superior electrochemical performance [5,12]. These results confirm that the synergistic effect of g-C_3_N_4_ and PMTB promotes rapid electron transfer and excellent electrocatalytic activity, making the nanohybrid-modified electrode highly suitable for sensitive electrochemical sensing applications.

#### 2.4.2. Cyclic Voltammetry Studies of Dopamine

The enhanced electrocatalytic behavior of the in situ fabricated g-C_3_N_4_/PMTB/SPCE toward the selective detection of DA was evaluated using CV. Figure 3b shows the CV responses obtained for DA (220 µM) in 0.1 M phosphate buffer (PB, pH 7.0). The SPCE (curve a) exhibits a weak DA oxidation peak at an anodic peak potential (*E*_pa_) of 0.24 V with a low anodic peak current (*I*_pa_) of 15.34 µA, and a corresponding cathodic peak at *E*_pc_ of 0.20 V with a cathodic peak current (*I*_pc_) of 10.13 µA. In comparison, the g-C_3_N_4_/SPCE (curve b) shows improved electrocatalytic performance, with DA oxidation occurring at a lower *E*_pa_ of 0.19 V and an increased *I*_pa_ of 18.29 µA, while the reduction peak appears at *E*_pc_ of 0.13 V with *I*_pc_ of 9.31 µA. Notably, the g-C_3_N_4_/PMTB/SPCE nanohybrid (curve c) demonstrates superior electrocatalytic activity toward DA oxidation, with a further reduced *E*_pa_ of 0.17 V and a nearly twofold enhancement in anodic peak current (*I*_pa_ = 30.44 µA), along with a cathodic peak at *E*_pc_ of 0.12 V and *I*_pc_ of 12.98 µA [5,6,21]. These results clearly indicate the synergistic effect of g-C_3_N_4_ and PMTB in facilitating electron transfer and lowering the overpotential for DA oxidation. Furthermore, the g-C_3_N_4_/PMTB/SPCE was employed for the electrocatalytic oxidation of DA over a wide concentration range. Figure 3c presents the CV responses recorded at various DA concentrations, while the corresponding calibration plot is shown in Figure 3d. A good linear relationship was obtained over the concentration range of 5–450 µM, with a low detection limit of 0.06 µM and a calculated sensitivity of 3.73 µA µM^−1^cm^−2^.

#### 2.4.3. Influence of pH Towards DA Oxidation

**Figure 4:**
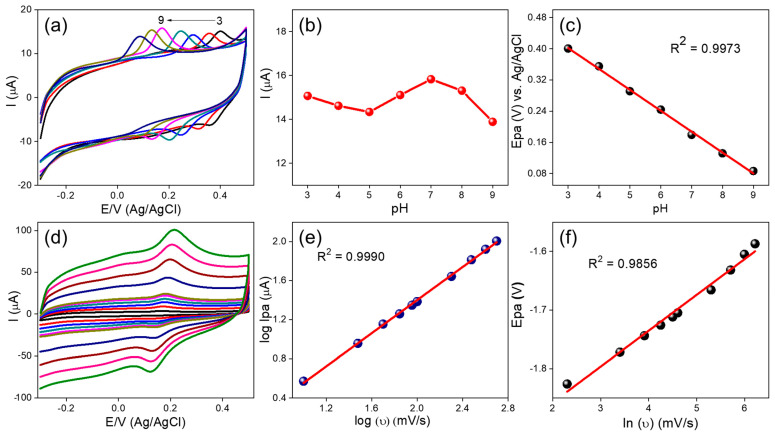
(**a**) Influence of different pH condition in 40 µM of DA (**b**) pH vs. current, (**c**) pH vs. potential shift plot, (**d**) Influence of different scan rate (10 to 500 mV/s) in 104 µM of DA (**e**) plots of log (ν) vs. log I_pa_ and (**f**) relationship of the peak potential (E_p_) vs. ln (ν).

The electrochemical oxidation behavior of DA at the g-C_3_N_4_/PMTB/SPCE was investigated under different pH conditions. Figure 4a shows the cyclic voltammograms recorded for 40 µM DA in phosphate-buffered solutions with pH values ranging from 3 to 9. As the pH increased (Figure 4b), the DA oxidation peak potential gradually shifted toward more negative (cathodic) potentials, indicating the involvement of protons in the electrochemical oxidation process [22]. The variation of the DA oxidation peak potential with pH is plotted in Figure 4c, showing a good linear relationship described by the equation *E*_pa_ = 0.053 pH + 0.562 (R^2^ = 0.9973). This linear dependence of oxidation potential on pH suggests that the electrochemical oxidation of DA follows a proton-coupled electron transfer mechanism. The observed slope is consistent with the Nernst equation, confirming the participation of both electrons and protons in the DA redox process.

dEp/dpH=2.303mRT/nF

**Figure 5:**
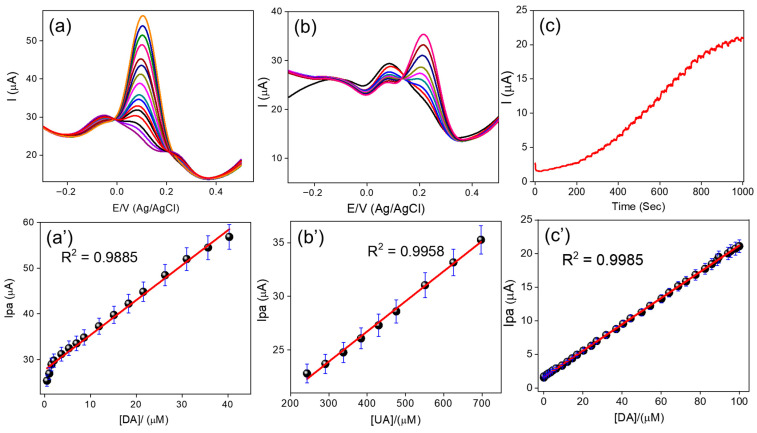
(**a**) DPV response of DA in the presence of 550 µM of UA and 900 µM of AA, DA concentration ranges from 0.05 µM to 40 µM (**a’**) concentration vs. current plot of DA. (**b**) DPV response of UA in the presence of 3 µM of DA and 900 µM AA, UA concentration range from 243 µM–697 µM and corresponding calibration plot (**b’**), (**c**) amperometry i-t curve response of DA detection from 0.01 to 100 µM and calibration plot (**c’**).

Here, *m* and *n* represent the number of protons and electrons involved in the oxidation process, respectively, while *R*, *T*, and *F* have their conventional meanings. Based on the obtained slope value, the oxidation of DA is confirmed to involve a two-electron/two-proton (2e^−^/2H^+^) transfer process. Consequently, the ratio of electrons to protons participating in the DA oxidation reaction is 1:1, which is consistent with previously reported results. Furthermore, the highest anodic peak current for DA oxidation was observed at pH 7.0 (Figure 5b). Therefore, pH 7.0 was selected as the optimal supporting electrolyte condition for all subsequent electrochemical experiments involving DA detection.

#### 2.4.4. Influence of Scan Rate Towards DA Oxidation

The influence of scan rates on DA detection at the g-C_3_N_4_/PMTB/SPCE was investigated using CV over a range of 10–500 mV/s (Figure 4d). Figure 4e shows the double-logarithmic plot of log ν versus log *I*_pa_, which exhibits a good linear relationship described by the regression equation log *I*_pa_ = 1.9797 log ν + 0.0611 (R^2^ = 0.9990). The near-unity slope indicates that the electron transfer process is predominantly adsorption-controlled [21]. The relationship between the anodic peak potential (*E*_pa_) and ln ν is presented in Figure 4f. As the scan rate increases, *E*_pa_ shifts linearly toward more positive potentials, following the regression equation *E*_pa_ = 0.2912 ln ν + 0.8463 (R^2^ = 0.9856) over the scan rate range of 10–500 mV/s. This dependence of *E*_pa_ on ln ν can be interpreted using the Laviron equation [27]:

$$E_{pa}\;=\;E^{0^{\prime }}\;+\;\frac{RT}{(1-\alpha )nF}\ln\left(\frac{RTk_{s}}{\left(1-\alpha \right)nF}\right)\;+\;\frac{RT}{(1-\alpha )nF}ln\nu$$

where α is the electron transfer coefficient, k_s_ is the standard heterogeneous rate constant, n is the number of electrons transferred, and R, T, and F have their usual meanings. From the slope of the *E*_pa_ versus ln ν plot, the electron transfer coefficient (α) was calculated to be 0.930, while the standard rate constant (k_s_) was estimated to be 1.346 s^−1^ for the electrochemical oxidation of DA at the g-C_3_N_4_/PMTB/SPCE [6]. These results further confirm the rapid electron transfer kinetics and excellent electrocatalytic performance of the fabricated nanohybrid electrode.

#### 2.4.5. DPV Studies of Selective Oxidation of DA in the Occurrence of AA and UA

Figure 5a shows the differential pulse voltammetry (DPV) response for the selective oxidation of D) in the presence of 900 µM AA and 550 µM UA. The measurements were performed in 0.1 M phosphate buffer (PB, pH 7.0) as the supporting electrolyte. Under neutral pH conditions, the carboxylic acid (-COOH) groups of the in situ electrodeposited g-C_3_N_4_/PMTB/SPCE are deprotonated to form negatively charged carboxylate (-COO^−^) groups, as the pK_a_ of the carboxylic acid moiety lies between 4 and 5. In contrast, dopamine, with a pK_a_ value of 8.9 for the amine group, exists predominantly in its protonated, positively charged form (-NH_3_^+^) at pH 7.0 [10,21]. Consequently, strong electrostatic attraction is established between the negatively charged -COO^−^ groups on the g-C_3_N_4_/PMTB/SPCE surface and the positively charged DA molecules, facilitating selective DA oxidation.

Figure 5a’ presents the corresponding calibration plot for DA, showing a linear concentration range from 0.05 to 40 µM, with a regression equation of *I*_pa_ (µA) = 0.7621C + 2.7768 (R^2^ = 0.9885). The LOD, calculated using the standard equation LOD = 3σ/q, was found to be 0.001 µM, and the sensitivity of the electrode was estimated as 1.85 µA µM^−1^ cm^−2^. The excellent electrochemical performance of the g-C_3_N_4_/PMTB/SPCE can be attributed to multiple synergistic interactions, including electrostatic attraction between DA and surface -COO^−^ groups, hydrogen bonding interactions, and π–π stacking between the hexagonal aromatic framework of the g-C_3_N_4_/PMTB nanohybrid and the phenyl ring of DA [5,6]. These interactions collectively enhance the selective oxidation of DA. In contrast, UA exhibits oxidation only at significantly higher concentrations and with lower sensitivity, which can be attributed to electrostatic repulsion between the negatively charged UA species and the -COO^−^ groups on the g-C_3_N_4_/PMTB/SPCE surface.

Figure 5b illustrates the DPV response of 3 µM DA in 0.1 M PB (pH 7.0) in the presence of increasing concentrations of UA. The corresponding calibration plot for UA oxidation (Figure 5b’) shows a linear concentration range from 243 to 697 µM, described by the equation *I*_p_ = 0.0282C + 1.5484 (R^2^ = 0.9958), with a relatively high LOD of 81 µM. Compared to DA, the required UA concentration is approximately 200 times higher, which is consistent with its lower pK_a_ value (3.7) and the resulting electrostatic repulsion between UA and the negatively charged g-C_3_N_4_/PMTB/SPCE surface. Furthermore, Figure S2 shows the DPV response of 0.5 µM DA and 243 µM UA in 0.1 M PB (pH 7.0) upon the addition of various concentrations of AA. No significant oxidation peak for AA was observed, which can be attributed to strong electrostatic repulsion between the negatively charged AA molecules and the g-C_3_N_4_/PMTB/SPCE surface, as well as π-π repulsion between AA and the benzene moieties of PMTB. This behavior is consistent with the pK_a_ value of AA (4.1), further confirming the excellent selectivity of the proposed sensing platform.

#### 2.4.6. Chronoamperometric i-t Curve Studies of DA Oxidation

The g-C_3_N_4_/PMTB/SPCE nanohybrid was successfully applied for DA oxidation under hydrodynamic flow conditions. The hydrodynamic voltammetric response of the g-C_3_N_4_/PMTB/SPCE is shown in Figure S3, where the DA oxidation current was recorded over a potential range of −0.2 to 0.6 V under continuous stirring. From the hydrodynamic voltammograms, the g-C_3_N_4_/PMTB/SPCE exhibited a maximum oxidation current for 30 µM DA at a constant potential of 0.2 V. Accordingly, 0.2 V was selected as the optimal working potential for DA oxidation at the g-C_3_N_4_/PMTB/SPCE, and this potential was subsequently fixed for amperometric detection using the current-time (i-t) technique. The g-C_3_N_4_/PMTB/SPCE was further employed in chronoamperometric experiments, where it demonstrated excellent oxidation performance compared with other electrochemical techniques. Figure 5c shows the chronoamperometric i-t responses obtained upon successive additions of different DA concentrations. The corresponding calibration plot of oxidation current (*I*_pa_) versus DA concentration is presented in Figure 5c’. A good linear relationship was obtained over the concentration range of 0.01–100 µM, described by the regression equation *I*_pa_ = 1.6150C + 0.1957 (R^2^ = 0.9985). The LOD was calculated to be 0.0035 µM, and the sensitivity was estimated as 9.74 µA µM^−1^ cm^−2^.

Furthermore, the analytical performance of the g-C_3_N_4_/PMTB/SPCE was compared with previously reported electrode materials, and the corresponding analytical parameters are summarized in Table 1. In particular, the oxidation performance of the proposed sensor was evaluated not only against glassy carbon electrode (GCE) based systems, but also in comparison with various modified working electrodes, including carbon-based nanomaterial electrodes and polymer film modified electrodes. These reported sensors, employing different electrochemical techniques such as DPV, square-wave voltammetry (SWV), and i-t, were systematically compared with the proposed g-C_3_N_4_/PMTB/SPCE, highlighting its superior analytical performance.

#### 2.4.7. Estimation of Dopamine in Biofluid Samples

Finally, the proposed g-C_3_N_4_/PMTB/SPCE was successfully applied to the determination of DA in real human biofluid samples. The biofluid sample preparation procedures are described in detail in the Experimental Section (Section 2.2), and the obtained DPV results are summarized in Table S1. The DA concentrations determined using the proposed nanohybrid-modified electrode were further validated by comparison with HPLC measurements. The close agreement between the electrochemical and HPLC results, along with excellent recovery values, demonstrates the accuracy and reliability of the developed sensor. The determination of DA in various human biofluids with satisfactory recovery confirms the real-world applicability of the g-C_3_N_4_/PMTB/SPCE. A key significance of the proposed sensing platform lies in the first-time in situ fabrication of a nanohybrid composed of polymethyl thymol blue and g-C_3_N_4_ on a SPCE. This fabrication strategy is considerably simpler than many previously reported electrode modification methods [5,6]. Moreover, the proposed electrode offers several practical advantages, including ease of use, low cost, and environmental friendliness. In addition, the g-C_3_N_4_/PMTB nanohybrid provides a large electrochemically active surface area and excellent electrocatalytic activity, making it a highly promising platform for selective dopamine sensing in practical biomedical applications.

## 3. Conclusions

The fabricated g-C_3_N_4_/PMTB/SPCE exhibits high selectivity and sensitivity toward the oxidation of DA in the presence of AA and UA. The proposed g-C_3_N_4_/PMTB nanohybrid acts as an efficient electrocatalyst due to its large electrochemically active surface area and excellent electron-transfer properties. The selective oxidation of DA in the presence of AA and UA was systematically investigated using DPV and amperometric i-t techniques. The corresponding analytical parameters, including the linear concentration range, LOD, and sensitivity for DA detection, are summarized in Table S2. Moreover, the developed g-C_3_N_4_/PMTB/SPCE offers several practical advantages, such as low fabrication cost, environmental friendliness, ease of use, long-term stability, and excellent reproducibility. Finally, the sensor was successfully applied to the determination of DA in real human biofluid samples, including saliva, blood serum, and urine, yielding satisfactory recovery results. These findings confirm the strong potential of the proposed g-C_3_N_4_/PMTB/SPCE as a reliable and practical platform for selective DA detection in biomedical applications.

## Figures and Tables

**Table 1:** Comparison of analytical parameters of DA analysis of in situ coated g-C_3_N_4_/PMTB/SPCE with other modified electrodes.

Electrode Materials	Linear Range (µM)	LOD (µM)	Sensitivity (µA/µM·cm^−2^)	Technique	Ref
PEDOT-PANS/GCE	2–100	0.5	-	LSV	[28]
Poly (Gallic acid)/GCE	5–100	3.6	-	DPV	[27]
Nafion/GCE	10–100	3.0	-	CV	[29]
Poly 2-napthol orange/PIGE	0.6–250	0.13	-	DPV	[21]
CuTRZMoO_4_/PPynanocomposite	1–100	0.080	-	DPV	[30]
Graphene-GQDs	0.1–100	0.03	14.25	DPV	[31]
Ni/rGO-oxCNF	10–16	0.6	1.64	DPV	[32]
PDA-CG/Pt	0.2–63	0.048	-	Amp	[33]
AgPd@Zr-MOF	2–42	0.1	10.26	SWV	[34]
CoP@C/NCS	0.2–12 5–400	0.03 1.4	9.4 0.04	SWV Amp	[35]
Poly (evans blue)/GCE	1–30	0.25	-	DPV	[36]
rGO-ZrO_2_/GCE	0.01–1000	0.0063	-	DPV	[37]
Cu-MIL-88B(Fe)/rGO/GCE	0.02–20	0.0076	-	DPV	[38]
MWCNT/Lac/GCE	0.1–6.0	-	-	SWV	[39]
g-C_3_N_4_/PMTB/SPCE	5–450 0.05–40 0.01–100	0.06 0.001 0.0035	3.73 1.85 9.74	CV DPV Amp	This work

## Data Availability

The original contributions presented in this study are included in the article. Further inquiries can be directed at the corresponding authors.

## Supplementary Materials

The following supporting information can be downloaded at: https://www.mdpi.com/article/10.3390/bios16020124/s1. Figure S1. Cyclic voltammograms of electrochemical polymerization/deposition of g-C_3_N_4_/methyl thymol blue in the potential of −0.4 V to 0.8 V with a scan rate of 100 mV s^−1^ in 0.5 M H_2_SO_4_ as a supporting electrolyte and the sweeping segments of 30 cycles. (The inset figures are two red-ox couples with zoom view). Scheme S1. Proposed mechanism for methyl thymol blue polymerization. Figure S2. DPV response of g-C_3_N_4_/PMTB/SPCE in different concentration AA and fixed concentration of 550 µM of UA and 3 µM of DA. Figure S3. Hydrodynamic voltametric response g-C_3_N_4_/PMTB/SPCE of 2.5 μM of DA in 0.1 M PB solution under stirring conditions. Table S1. Detection of DA in different human biofluid samples (n = 3). Table S2. The fabricated g-C_3_N_4_/PMTB/SPCE for DA detection by using different electrochemical techniques and corresponding analytical parameters [17,27,40,41].
