# Supplementary material for: Metal-Free Electrochemical Dopamine Sensing Using a g-C3N4/Polymethyl Thymol Blue Nanohybrid
Source: Biosensors (Basel). 2026 Feb 17;16(2):124. doi: 10.3390/bios16020124 (PMC12937753; doi:10.3390/bios16020124)
Supplement: Supplementary file 1 [file biosensors-16-00124-s001.zip › biosensors-4128716-supplementary.pdf]

Supporting Information

Metal-Free Electrochemical Dopamine Sensing Using a g-C_3N_4/Polymethyl Thymol Blue Nanohybrid

Sankar Sekar [1,2], Sejoon Lee [1,2,*], Sutha Sadhasivam [3], Kumar Sangeetha Selvan [4], Saravanan Sekar [5], Youngmin Lee [1,2], Pugazhendi Ilanchezhiyan [6,7], Seung-Cheol Chang [8,*] and Ramalingam Manikandan [8,9,*]

1 Division of System Semiconductor, Dongguk University-Seoul, Seoul 04620, Republic of Korea
2 Quantum-Functional Semiconductor Research Center, Dongguk University-Seoul, Seoul 04620, Republic of Korea
3 Department of Chemistry, CMS College of Engineering, Namakkal 637003, Tamil Nadu, India
4 Department of Chemistry, VelTech Rangarajan Dr. Sagunthala R&D Institute of Science and Technology, Avadi, Chennai 600062, Tamil Nadu, India
5 Department of Mechanical Engineering, K. Ramakrishnan College of Technology, Trichy 621112, Tamil Nadu, India
6 Centre for Research and Development, Sri Manakula Vinayagar Engineering College, Madagadipet, Puducherry 605107, India
7 Department of Physics, Sri Manakula Vinayagar Engineering College, Madagadipet, Puducherry 605107, India
8 Department of Cogno-Mechatronics Engineering, College of Nanoscience and Nanotechnology, Pusan National University, Busan 46241, Republic of Korea
9 Engineering Research Center for Color-Modulated Extra-Sensory Perception Technology, Pusan National University, Busan 46241, Republic of Korea
* Correspondence: sejoon@dongguk.edu (S.L.); s.c.chang@pusan.ac.kr (S.-C.C.); maniiichem@gmail.com (R.M.)

S1. Materials and Methods

S1.1 Reagents and Instrumentation

Melamine, methyl thymol blue, ascorbic acid, dopamine, uric acid is A.R reagents were obtained from Sinopharm Reagents Company. $K_3[Fe (CN)6]$, KH_2PO_4, K_2HPO_4, CH_3COONa, and CH_3COOH were brought from Macklin Biochemicals. H_2SO_4 (98%), HCl (37%), NaOH and KCl were purchased from Merk company. Working screen-printed carbon electrode with working area of 0.07 cm^2 was brought from Zensor research and development, Taiwan. The entire supporting electrolytes, and biomolecules have been prepared by de-ionized water (18.2 MΩ/cm) and the entire electroanalytical tests were performed at ambient conditions (26ºC ±1). Finally, all the reagents were utilized as brought without extra purification.

The morphology of the fabricated nanohybrid material has been captured by using FE-SEM coupled with EDX SU6600 (Hitachi, Japan). The functional group vibrations and structures of the materials were examined by Thermo Scientific Nicolet iS50 (Japan) FT-IR spectrometer. The crystalline pattern of the nanohybrid has been confirmed by Rigaku smart lab X-ray spectrometer (XRD) with a working voltage of 40 kV and Cu Kα of 15406 nm. XPS studies were carried out by JEOL J9200 photoelectron spectrometer (Japan) for the elemental composition of the nanohybrid. The electrochemical investigations are performed by CHI 660B electrochemical analyzer. The typical three electrode setup electrochemical cell containing, in-situ coated g-C_3N_4/PMTB on the screen-printed carbon electrode as active (working) electrode, Ag/AgCl, and Pt wire acted as a reference and auxiliary electrode respectively. The entire supporting electrolyte solutions were purged with N_2 gas for three mins previous commitment of the electrochemical investigations. In the entire electrochemical measurements average of four replicate measurements was performed, and appropriate calibration plots with error bars.

S1.2 Preparation of biofluid samples

The practical applicability of the prepared nanohybrid electrode was examined using DA in different biofluid samples. The major interfering species present in biofluids such as AA and UA, were

selected to evaluate the selective sensing performance toward DA. Briefly, 20 mL of 0.1 M phosphate buffer (PB) solution was used as the supporting electrolyte for selective DA detection. A fixed concentration of AA and UA, along with varying concentrations of DA, was introduced into the electrolyte, and the corresponding oxidation current of DA was recorded using the DPV technique [1,2]. The detection of DA was further investigated in synthetic and commercial biofluid samples, including commercial blood serum and artificial urine. The commercial serum samples were directly purchased from Sigma-Aldrich and diluted appropriately with 0.1 M PB solution prior to analysis, following our earlier report. In addition, artificial urine was prepared to simulate the ionic composition and pH of human urine, containing major inorganic salts (e.g., Na^+, K^+, Cl^-, phosphate species), urea as the dominant metabolite, and representative endogenous interferents such as creatinine and UA. The pH of the artificial urine was adjusted to the physiological urinary range (≈6.0-6.5) before electrochemical measurements.

The prepared synthetic urine sample was diluted with PB solution and employed for electrochemical detection of DA under identical DPV conditions. The human biofluid samples exhibited a clear oxidation current response corresponding to DA. For quantitative real sample analysis, a known volume of DA standard solution was spiked into the biofluid matrix, and the recovery was evaluated using the standard addition method.

S1.3 g-C_3N_4 preparation

g-C_3N_4 has been prepared through a hydrothermal process by using melamine as a precursor. About 1g of melamine was taken in a silica crucible covered with a lid, followed by the crucible was kept in the high-temperature muffle furnace under the inert (bypassing N_2 gas) atmosphere at a heating temperature of 5°C/min up to 550°C for 2 hours. Afterward, the silica crucible was carefully taken in the furnace and allowed to reach atmospheric condition and the obtained yellow color fine powder. The final yellow color powder material (g-C_3N_4) has been washed with de-ionized water and ethanol under the

ultrasonic process to attain a g-C_3N_4 nanomaterial[3]. Further, the g-C_3N_4 has been dried in a vacuum oven at 65°C overnight and stored in an airtight container for upcoming experiments.

S1.4 SPCE pre-anodization procedure

To remove the electrode debris and other impurities on the electrode surface, the electrode was cleaned by using de-ionized water and ethanol before committing the electrochemical experiments. The pre-anodization procedure has been performed by using CV in 0.1 M of acetate buffer with the sweeping potential of -0.1 to 2 V for 30 segments. As prepared activated (pre-anodized) electrode was cleaned/rinsed with de-ionized water followed by drying at dust-less vacuum oven at 40°C temperature for 6 hours[4]. Followed by the pretreated SPCE was utilized for the electrochemical deposition/polymerization of nanohybrid material followed by selective electrochemical sensing of neurotransmitters in biofluids.

S1.5 In-situ single step electrochemical deposition/polymerization of g-C_3N_4/PMTB nanohybrid

As prepared g-C_3N_4/PMTB nanohybrid was carried out by electrochemical polymerization/deposition method. An electrochemical cell containing 10 mg of g-C_3N_4 fine powder and 0.5 mM of methyl thymol blue in 0.5 M of H_2SO_4 as supporting electrolyte under ultrasonication for 2 hours. The pre-anodized (activated) SPCE were dipped in the brownish- yellow colour electrolyte and the cyclic voltammogram was recorded in the operating potential of -0.4 to 0.8 V at 100 mV/s scan rate for 30 successful sweeping segments. After that g-C_3N_4 as well as a monomer of the methyl thymol blue was deposited as a polymerized nanohybrid like a network structured form on the SPCE surface. This electrode was hereafter denoted as g-C_3N_4/PMTB/SPCE and the fabricated electrode was kept in the air-tight container under dried condition for further experiments.

S1.6 Influence of other species

The main problem of the prepared g-C_3N_4/PMTB/SPCE towards selective analysis of DA, UA, and AA is the most influencing species in the biofluids. The selective sensing of DA in occurrence of AA and UA were investigated by DPV method and the reason was discussed. The other interfering foreign

substances (20-fold excess concentration of each analyte) like glucose, sucrose, glycine, cysteine, folic acid, citric acid, oxalic acid, caffeic acid, urea, serotonin, L-tyrosine, norepinephrine, epinephrine, and gallic acid were tested in the presence of DA, the amperometric response of these interfering species does not affect/detect the analyte and the obtained results are shown in **fig-S4a**. These analytes were selected based on the nearby oxidation potential of DA. Further, the metals ions (50-fold excess concentration) like K^+, Na^+, Ca^{2+}, Mg^{2+}, NH_4^+, Al^{3+}, Ba^{2+}, and the anions like Cl^-, Br^-, F, NO_3^-, SO_4^{2-} and CO_3^{2-} were added towards oxidation of DA, and these species did not interfere the oxidation current of DA and the obtained results are shown in **fig-S4b**. These metal ions and cations are normally voltammetric inactive.

S1.7 Reproducibility and stability studies

As prepared g-C_3N_4/PMTB/SPCE was examined for the reproducibility towards DA oxidation has been tested with four different kinds of electrodes towards detection of similar DA concentration. The oxidation current response of all four electrodes was nearly similar with the calculated relative deviation of 0.73%. Further, the stability of the proposed electrode towards DA oxidation was tested by using cyclic voltammetry in the time duration of every five hours, followed by over 90 days, the electrode has shown stable oxidation current of 33.5 μA (20 μM of DA) and the outputs are displayed in **Fig-S4c & d.** The g-C_3N_4/PMTB/SPCE has shown 97.83% of original oxidation current and the found RSD value of 0.71% which is denoted that the prepared electrode has good long-term stability towards analytes detection. After the experiments, the g-C_3N_4/PMTB/SPCE was kept in the air-tight container at atmospheric for long-term use.

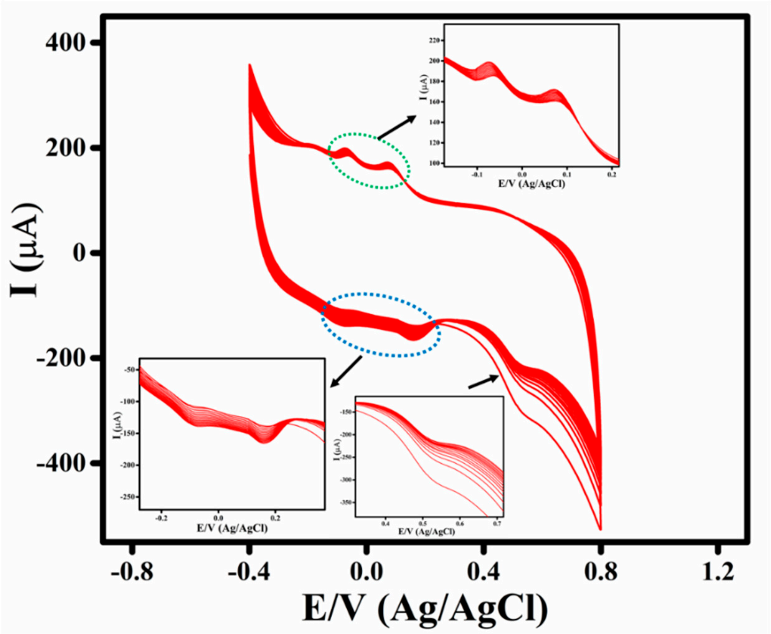

Fig-S1. Cyclic voltammograms of electrochemical polymerization/deposition of g-C_3N_4/methyl thymol blue in the potential of -0.4 V to 0.8 V with a scan rate of 100 mV s^{-1} in 0.5 M H_2SO_4 as a supporting electrolyte and the sweeping segments of 30 cycles. (The inset figures are two red-ox couples with zoom view).

Scheme-S1 Proposed mechanism for methyl thymol blue polymerization

Step-1 Formation of free radicals

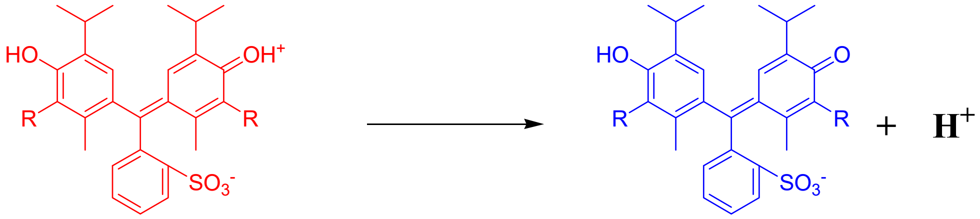

[A] [B]

Step-2 Free radical's initiation

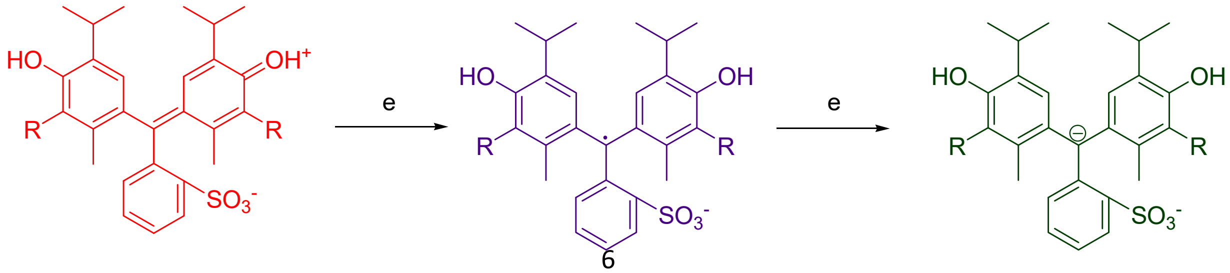

[A] [C] [D]

Step-3

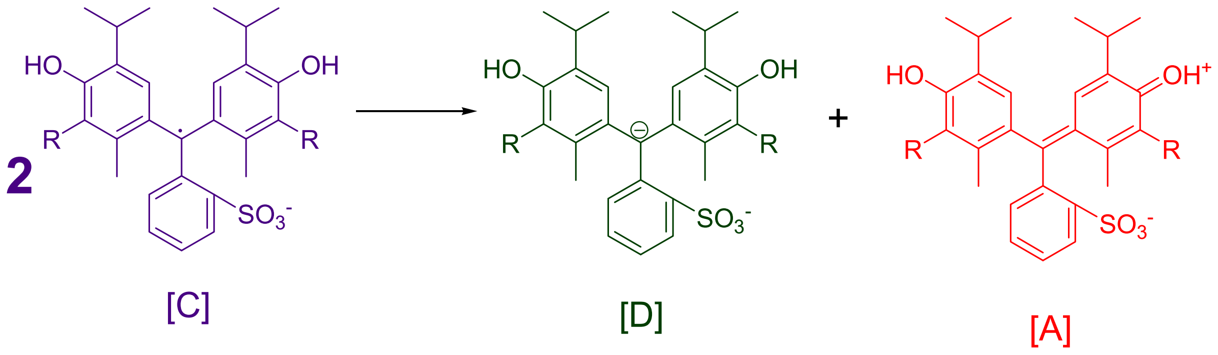

Step-4

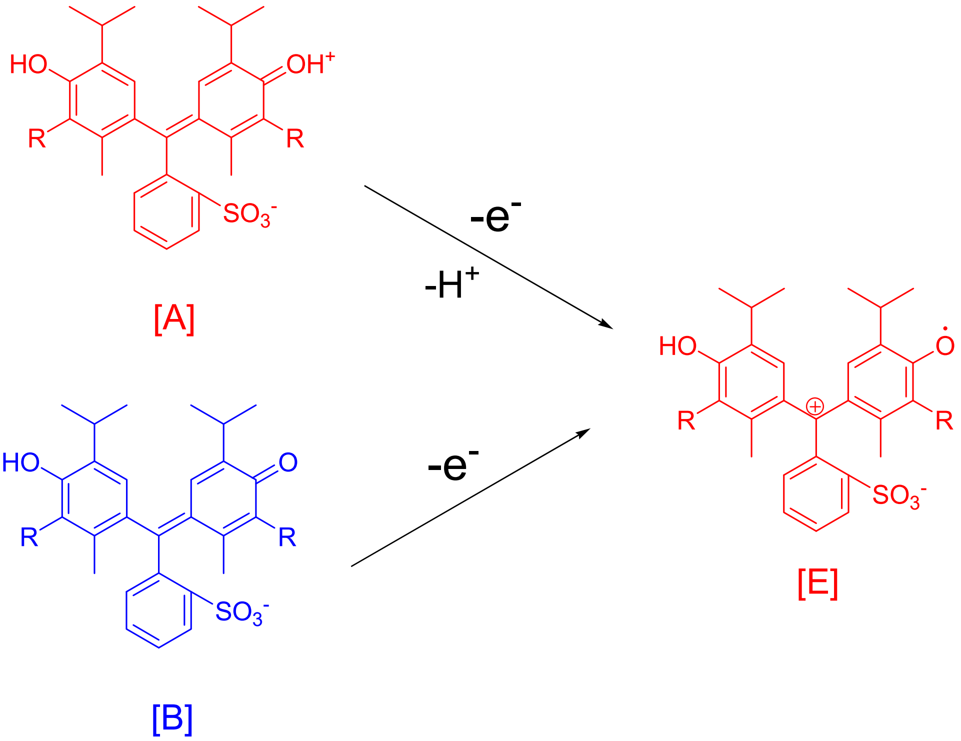

Step-5 Polymer film formation

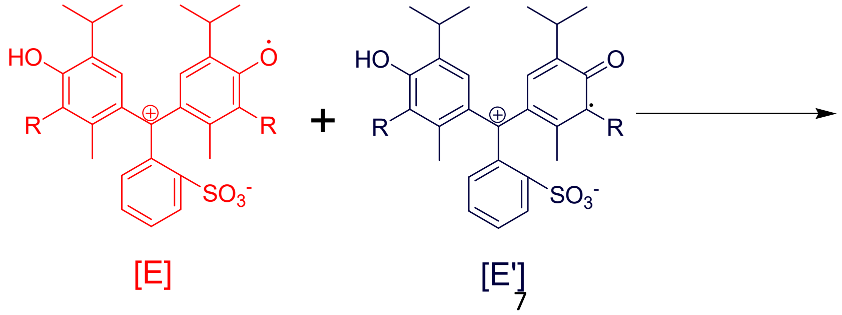

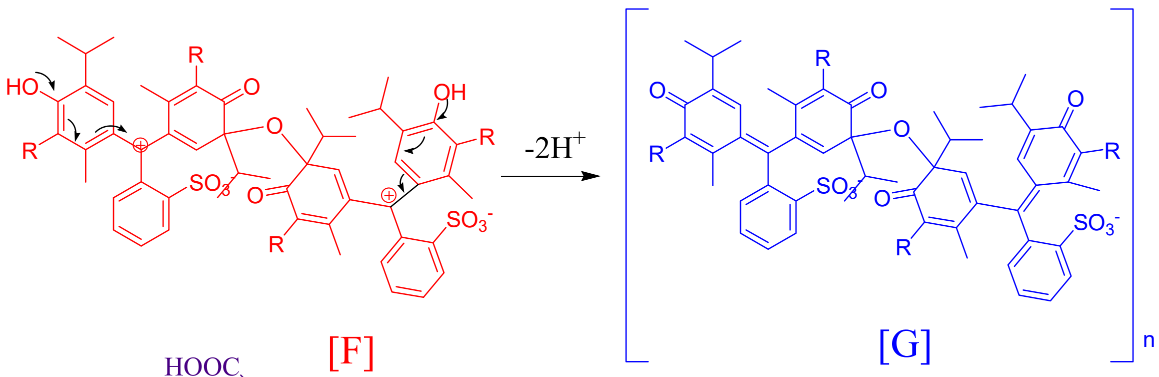
-2H+
[F]
[G]
n

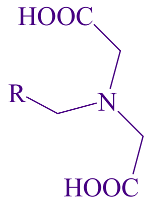
HOOC
R
N
HOOC

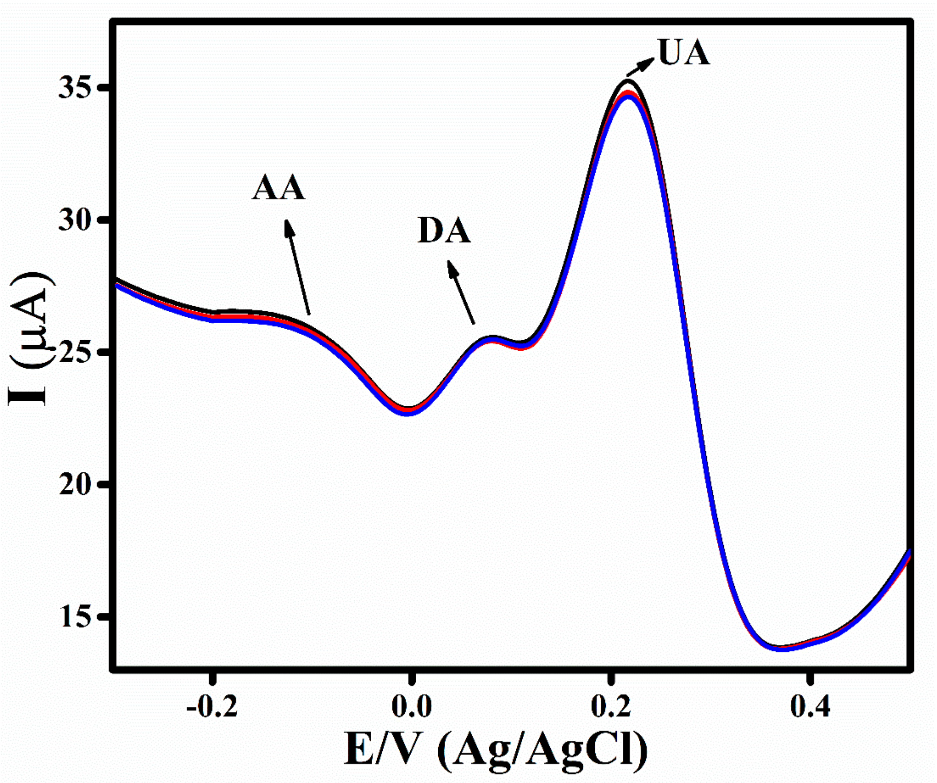

Fig-S2. DPV response of g-C_3N_4/PMTB/SPCE in different concentration AA and fixed concentration of 550 μM of UA and 3 μM of DA.

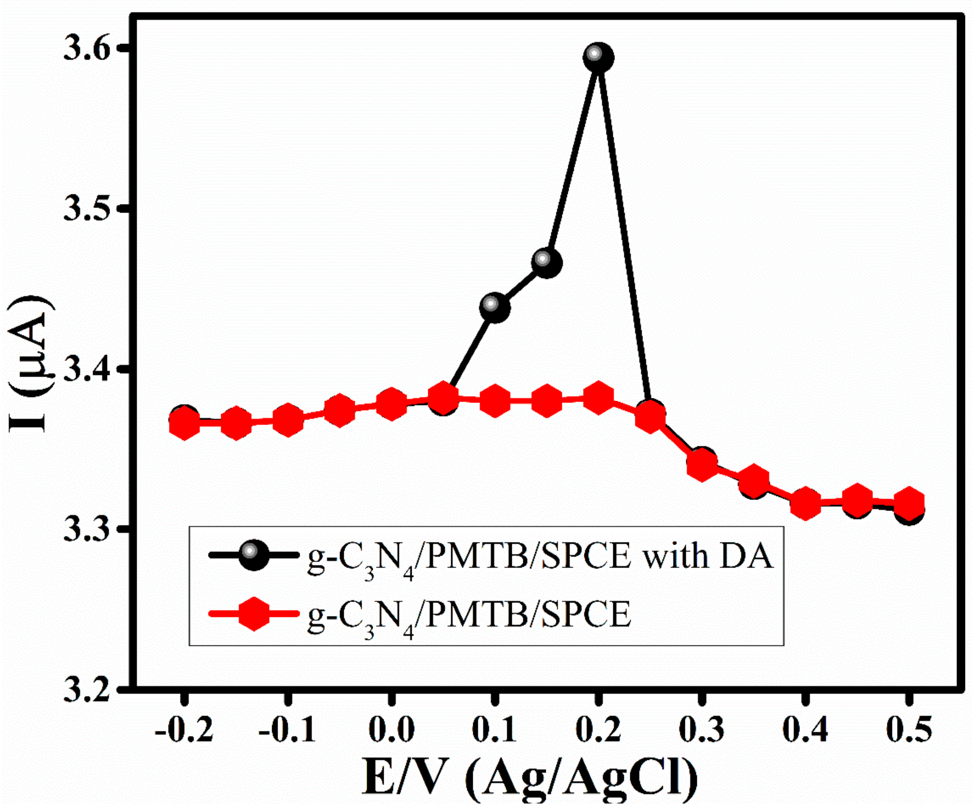

Fig-S3. Hydrodynamic voltametric response g-C_3N_4/PMTB/SPCE of 2.5 μM of DA in 0.1 M PB solution under stirring conditions.

Table-T1 Detection of DA in different human biofluid samples (n=3).

Biofluid name	Detected (μM)	Spiked (μM)	Found (μM)	Recovery (%)
DPV Method Blood serum	0.05	5.0	5.09(±0.08)	100.79
	0.05	10.5	10.61(±0.11)	100.56
	0.71	20.35	21.01(±0.04)	99.76
Urine	0.55	10.5	11.10(±0.02)	100.45
	0.57	20.5	21.29(±0.08)	101.04
	0.60	30.3	31.03(±0.01)	100.42
HPLC Blood serum	0.08	5.0	5.12 (±0.12)	100.78
	0.1	10.5	10.63(±0.06)	100.28
	0.85	20.4	21.58(±0.04)	101.55
Urine	0.61	10.4	11.12(±0.001)	100.99
	0.59	20.5	22.31(±0.07)	105.78
	0.63	30.1	31.02(±0.03)	100.94

Table-T2 The fabricated g-C_3N_4/PMTB/SPCE for DA detection by using different electrochemical techniques and corresponding analytical parameters.

S.No	Techniques	Linear range (μM)	LOD (μM)	Sensitivity (μA. μM cm^{-2})
1.	Cyclic voltammetry (CV)	5 - 450	0.06	3.73
2.	Chronoamperometry (CA)	0.01-100	0.0035	9.74
3.	Differential pulse voltammetry (DPV)	0.05 - 40	0.001	1.85

References

1. Manikandan, R.; Deepa, P.; Narayanan, S.S. Fabrication and characterization of poly 2-napthol orange film modified electrode and its application to selective detection of dopamine. *Journal of Solid State Electrochemistry* **2017**, *21*, 3567-3578.
2. Shafi, P.M.; Joseph, N.; Karthik, R.; Shim, J.-J.; Bose, A.C.; Ganesh, V. Lemon juice-assisted synthesis of LaMnO3 perovskite nanoparticles for electrochemical detection of dopamine. *Microchemical Journal* **2021**, *164*, 105945.
3. Ramalingam, M.; Ponnusamy, V.K.; Sangilimuthu, S.N. A nanocomposite consisting of porous graphitic carbon nitride nanosheets and oxidized multiwalled carbon nanotubes for simultaneous stripping voltammetric determination of cadmium (II), mercury (II), lead (II) and zinc (II). *Microchimica Acta* **2019**, *186*, 1-10.
4. Eswaran, M.; Tsai, P.-C.; Wu, M.-T.; Ponnusamy, V.K. Novel nano-engineered environmental sensor based on polymelamine/graphitic-carbon nitride nanohybrid material for sensitive and simultaneous monitoring of toxic heavy metals. *Journal of Hazardous Materials* **2021**, *418*, 126267.
